# Bifidogenic and butyrogenic effects of young barely leaf extract in an in vitro human colonic microbiota model

**DOI:** 10.1186/s13568-019-0911-5

**Published:** 2019-11-13

**Authors:** Daisuke Sasaki, Kengo Sasaki, Yasushi Kadowaki, Yasuyuki Aotsuka, Akihiko Kondo

**Affiliations:** 1 Graduate School of Science, Technology and Innovation, Kobe University, 1-1 Rokkodai-cho, Nada-ku, Kobe, Hyogo 657-8501 Japan; 2 JPD Co., Ltd., 7-98 Kita-Itami, Itami-shi, Hyogo, 664-0831 Japan; 3 RIKEN Center for Sustainable Resource Science, 1-7-22 Suehiro-cho, Tsurumi-ku, Yokohama, Kanagawa 230-0045 Japan

**Keywords:** Young barely leaf extract, Intestinal microbiota, In vitro model culture system, *Bifidobacterium*, Butyrate

## Abstract

Young barley leaf extract (YBL) contains beneficial substances such as fructans, minerals, and vitamins. The effects of YBL administration on the human colonic microbiota and its production of metabolites were evaluated using an in vitro model culture system. Fermentations were started by inoculating fecal samples from nine healthy subjects, with or without 1.5% YBL. Bacterial 16S rRNA sequencing results confirmed that YBL administration significantly increased the relative abundances of bacteria related to the genus *Bifidobacterium* (*p *= 0.001, paired t-test) and those of the genera *Faecalibacterium*, *Roseburia*, Unclassified *Ruminococcaceae*, and *Lachnospira* (*p *= 0.013, *p *= 0.019, *p *= 0.028, and *p *= 0.034, respectively, paired t-test). Increased abundances of the latter genera corresponded to increased butyrate production in human colonic microbiota models following fermentation with 1.5% YBL, when compared to fermentation without 1.5% YBL (*p *= 0.006, Dunnett’s test). In addition, YBL administration significantly increased the production levels of amino acids such as lysine, glutamate, serine, threonine, alanine, isoleucine, leucine, valine, and phenylalanine. Therefore, our results showed the health-promoting bifidogenic and butyrogenic effects of YBL.

## Introduction

Barley (*Hordeum vulgare* L.) is a cereal crop that it widely distributed throughout the world, and barley grass is rich in functional ingredients (Zeng et al. 2018). Young barley leaf and its extract are components of a green-colored drink named “Aojiru” in Japan (Yamaura et al. 2012; Ikeguchi et al. 2014; Yamaura et al. 2015). Barley grass (including young green leaves and stems) possesses several pharmacological activities such as anti-cancer activity, anti-oxidant activity, and anti-inflammation activity, and pharmacological interest exists in using barley grass to treat chronic diseases (Lahouar et al. 2015). Young barley leaf extract (YBL) is a natural source of vitamins and minerals, and supplementation with YBL decreased plasma cholesterol levels in hyperlipidemic patients (Yu et al. 2003). In addition, barley leaves temporarily accumulate fructan, sucrose, and starch as stored carbohydrates, particularly during nitrogen starvation (Wang et al. 2000). Vandeputte et al. (2017) observed that consumption of an inulin-type fructan increased the relative abundances of *Bifidobacterium* species in the fecal microbiota. To date, the impact of YBL interventions on the human colonic microbiota has not yet been investigated in detail.

Recently, we developed an in vitro human colonic microbiota model using a batch fermentation system and human fecal inocula (named as the Kobe University Human Intestinal Microbiota Model [KUHIMM]), which maintained the diversity and overall number of bacterial species in fecal samples (Sasaki et al. 2018). Thus, the KUHIMM served as a convenient model for simulating and evaluating the effect of exogenous functional compounds, such as prebiotics, on the human colonic microbiota. In addition, the KUHIMM could reproduce the production of short-chain fatty acids (SCFAs) in the colon. For example, the KUHIMM was used to detect significantly lower butyrogenesis in samples from ulcerative colitis patients (Sasaki et al. 2019). The aim of this study was to assess the impact of YBL administration on the human colonic microbiota composition using the KUHIMM. To this end, we performed high-throughput, next-generation sequencing (NGS) of the bacterial 16S rRNA gene and compared the KUHIMM results obtained with or without YBL supplementation.

## Materials and methods

### YBL acquisition and composition

YBL was supplied by JPD Co., Ltd. (Hyogo, Japan). The manufacturer collected leaves from young barley plants (*Hordeum vulgare* L. var. *nudum* Hook; 20–35 cm in height) in the Oita Prefecture of Japan and extracted juice from the leaves to produce YBL as a spray-dried powder. The YBL contained carbohydrates (46.5%), proteins (29.0%), minerals (17.5%), water (3.4%), fat (0.6%), fructan (9.4 g/100 g), K (4014 mg/100 g), Na (775 mg/100 g), Ca (619 mg/100 g), P (523 mg/100 g), Mg (237 mg/100 g), Fe (15.9 mg/100 g), Mn (4.72 mg/100 g), Zn (2.41 mg/100 g), chlorophyll (938 mg/100 g), and superoxide dismutase (SOD; 7716 U/g).

### Human fecal sample collection from volunteers

Fecal samples were obtained from nine healthy Japanese human volunteers, who had not been treated with antibiotics for more than 2 months prior to the experiment. All participants were recruited according to the inclusion criteria, which comprised an age of the early twenties to the middle forties, being Japanese, a non-smoking status, and good health and physical condition. The exclusion criteria included significant clinical deviation from normal as determined by the investigators; a history or suspicion of having diabetes, liver disease, kidney disease, or a food allergy; or currently taking prebiotics, probiotics, or lipid-lowering medications.

Fecal samples were immediately collected with an anaerobic culture swab (212550 BD BBL Culture Swab; Becton, Dickinson and Company, Franklin Lakes, NJ, USA) and used within 24 h.

### Operation of the KUHIMM, with and without YBL

The KUHIMM was operated with or without added YBL, using a multi-channel fermenter (Bio Jr.8; ABLE, Tokyo, Japan), as described in detail previously (Takagi et al. 2016; Sasaki et al. 2018). The cultivations were initiated by inoculating a single fecal suspension (100 µL) into each vessel. During cultivation at 37 °C, the culture broth was stirred at 300 rpm with a magnetic stirrer and continuously purged with a filter-sterilized mixture of gas to maintain anaerobic conditions. To evaluate the effect of YBL, YBL powder was added into one of the vessels at a final concentration of 15 g/L (1.5% per 100 mL medium) prior to cultivation. A control vessel without YBL powder was also prepared. Aliquots (1 mL) of culture broth were sampled from the vessels at 48 h after initiating cultivation. Fecal and culture broth samples were stored at − 20 °C until use.

### Extraction of microbial genomic DNA

Microbial genomic DNA was extracted from suspended feces and culture broth from the KUHIMM at 48 h, as described previously (Takagi et al. 2016). Purified DNA was eluted into TE buffer (10 mM Tris HCl containing 1.0 mM ethylenediaminetetraacetic acid) and stored at − 20 °C until use.

### Illumina library generation

Bacterial 16S rRNA genes (V3–V4 region) were amplified using genomic DNA as the template and the primers S-D-Bact-0341-b-S-17 (5′-CCTACGGGNGGCWGCAG-3′) and S-D-Bact-0785-a-A-21 (5′-GACTACHVGGGTATCTAATCC-3′) (Klindworth et al. 2013), as described previously (Sasaki et al. 2017). Index primers (Nextera XT Index Kit; Illumina Inc., San Diego, CA, USA) overhanging the amplified sequences were added to the gene-specific sequences. Each polymerase chain reaction (PCR) was performed according to the manufacturer’s instructions. Amplicons were purified with AMPure XP DNA purification beads (Beckman Coulter, Brea, CA, USA) and eluted in 25 µL of 10 mM Tris (pH 8.5). Purified amplicons were quantified using an Agilent Bioanalyzer 2100 with DNA 1000 chips (Agilent Technology, Santa Clara, CA, USA) and a Qubit 2.0 instrument (Thermo Fisher Inc., Waltham, MA, USA), and pooled at equimolar concentrations (5 nM). The 16S rRNA genes and an internal control (PhiX control v3; Illumina) were subjected to paired-end sequencing using a MiSeq instrument (Illumina) and the MiSeq Reagent Kit, v3 (600 cycles; Illumina). The PhiX sequences were removed, and paired-end reads with Q scores ≥ 20 were joined using the MacQIIME software package, version 1.9.1 (Caporaso et al. 2010). The UCLUST algorithm (Edgar 2010) was used to cluster the filtered sequences into operational taxonomic unit (OTUs) based on a ≥ 97% similarity threshold. Chimeric sequences were checked and removed from the library using ChimeraSlayer (Haas et al. 2011). Representative sequences from each OTU were taxonomically classified via the GreenGenes taxonomic database, using the Ribosomal Database Project Classifier (Wang et al. 2007).

### Real-time PCR analysis

Real-time PCR was performed to quantify total bacterial growth during cultivation, using a LightCycler 96 system (Roche, Basel, Switzerland) with a primer set targeting all eubacteria (Matsuki et al. 2004; Rinttila et al. 2004). PCR amplification was performed as described previously (Takagi et al. 2016).

### Measurement of SCFA concentrations

Concentrations of SCFAs such as acetate, propionate, butyrate, lactate, and succinate were measured using a high-performance liquid chromatography (HPLC) instrument (Shimadzu Corporation, Kyoto, Japan) equipped with an Aminex HPX-87H column (Bio-Rad Laboratories, Inc., Hercules, CA, USA) and a RID-10A refractive index detector (Shimadzu Corporation). The HPLC instrument was operated at 65 °C using 5 mM H_2_SO_4_ as the mobile phase with a flow rate of 0.6 mL/min.

### Measurement of amino acid concentrations

Free amino acids were extracted from supernatants collected from culture broths after 48 h of fermentation, using a modified cold chloroform–methanol method (Putri et al. 2013). The water phase of the extract (700 µL) was dried under vacuum and stored at − 80 °C until further analysis (Bennett et al. 2008).

The dried extract samples were thawed on ice and derivatized at 30 °C for 90 min with 100 µL of 20 mg/mL methoxyamine hydrochloride in pyridine, after which 50 µL *N*-methyl-*N*-(trimethylsilyl) trifluoroacetamide (GL Sciences, Tokyo, Japan) (Lisec et al. 2006) was added, followed by incubation at 37 °C for 30 min. The derivatized samples (1 µL) were subjected to gas chromatography–quadrupole–mass spectrometry (GC–Q–MS) using a GCMSQP-2010 system (Shimadzu). The details of the GC–Q–MS operating conditions and procedures were described previously (Kato et al. 2012; Sasaki et al. 2014). Free amino acid concentrations were measured in triplicate.

### Bioinformatics and statistical analyses

The α-diversity value (Shannon–Wiener index) was calculated from OTU numbers using the MacQIIME software package (Caporaso et al. 2010). Principal coordinate analysis (PCoA) was conducted using the OTU information for each sample and calculated based on unweighted UniFrac distances (Lozupone and Knight 2005) using MacQIIME. A nonparametric paired t-test (the Kruskal–Wallis test) and Dunnett’s test were performed using Prism 8 (GraphPad Software, Inc., San Diego, CA) and/or JMP 13 software (SAS Institute Inc., Cary, NC, USA). *p *< 0.05 was considered to reflect a statistically significant difference.

### Data availability

All raw sequence data generated in this study were deposited on the MG-RAST server (Meyer et al. 2008) (http://metagenomics.anl.gov) in a file named “Single Batch Fermentation System Simulating Human Colonic Microbiota_Young Barley Leaf Extract” under Accession Numbers mgm4854581.3–mgm4854607.3. The datasets supporting the conclusions of this article are included within the article (and its additional file).

## Results

### The bacterial diversity in feces was maintained after adding 1.5% YBL

**Fig. 1:**
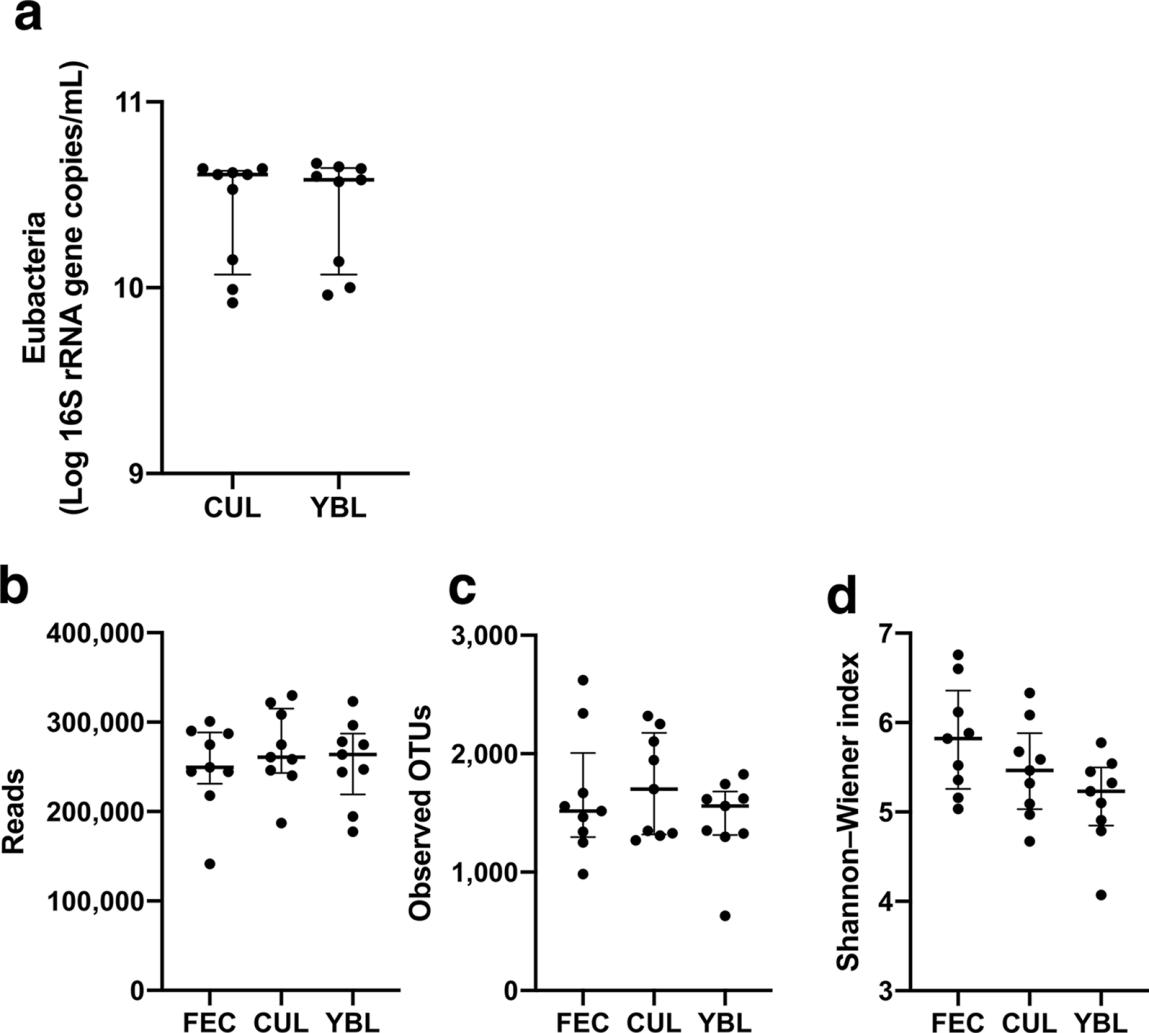
Summary of 16S rRNA gene data. 16S rRNA genes were amplified using DNA extracted from KUHIMM samples fermented for 48 h without 1.5% YBL (CUL) and with 1.5% YBL (YBL). **a** Eubacterial copy numbers. **b** Numbers of quality-controlled sequences obtained by NGS. **c** Observed numbers of OTUs. **d** Shannon–Wiener index. The data shown are presented as medians and interquartile ranges (25th–75th percentiles)

One of nine human fecal samples (designated as FEC in Fig. 1) was used as the inoculum, and the in vitro human colonic microbiota model, KUHIMM, was constructed with the addition of 1.5% YBL (designated as YBL in Fig. 1). Control cultures without added YBL were also prepared (designated as CUL in Fig. 1). Whole DNA was extracted from the original fecal samples and corresponding culture broths from the KUHIMM, with and without YBL, after 48 h of fermentation. The eubacterial copy numbers reached 0.83–4.73 × 10^10^ copies/mL using the KUHIMM at 48 h (Fig. 1a), which were comparable to the reported cell densities in human feces (approximately 10^11^ copies/g wet feces) (Sender et al. 2016). YBL administration did not affect the eubacterial copy numbers. In addition, bacterial 16S rRNA gene sequences were comprehensively analyzed by NGS. A total of 6977,688 quality-controlled reads were obtained (Fig. 1b). In terms of the OTU numbers (which define the sequence similarities among 16S rRNA gene-sequence clusters) and the Shannon–Wiener indexes of species diversity, no significant differences were observed between the FEC, CUL, and YBL samples (*p *> 0.05, Kruskal–Wallis test; Fig. 1b–d). Therefore, the bacterial diversity in the KUHIMM did not change following the addition of 1.5% YBL.

### YBL administration changed the microbiota composition

**Fig. 2:**
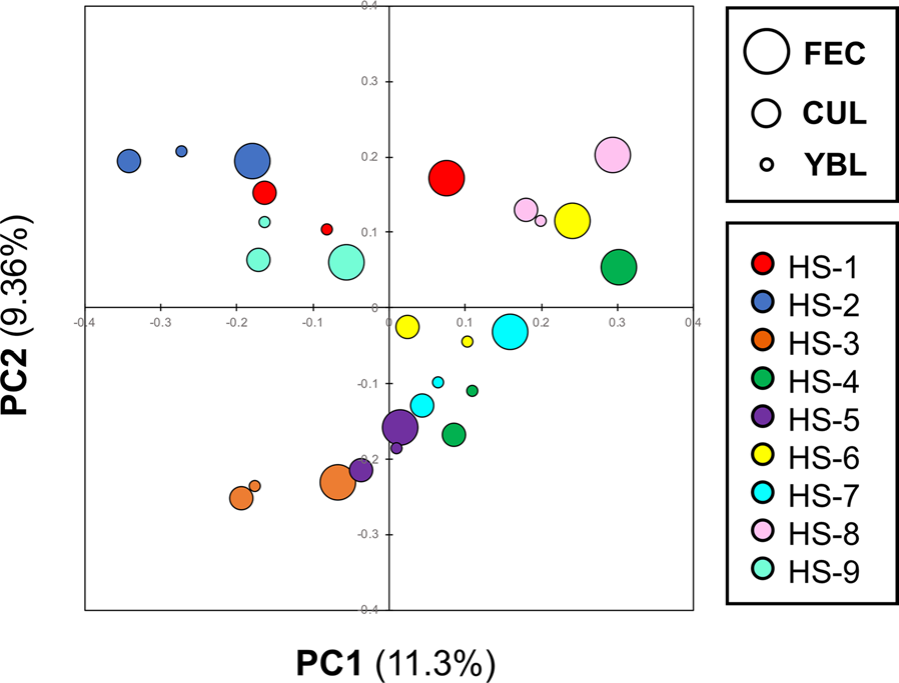
PCoA of 16S rRNA gene sequences from fecal samples of nine healthy subjects (HS-1–HS-9). The large, medium, and small circles in the PCoA plot represent the microbiota in feces, the corresponding KUHIMM cultures without YBL, and the KUHIMM cultures with 1.5% YBL, respectively. The cultures were sampled at 48 h after fermentation was initiated

**Fig. 3:**
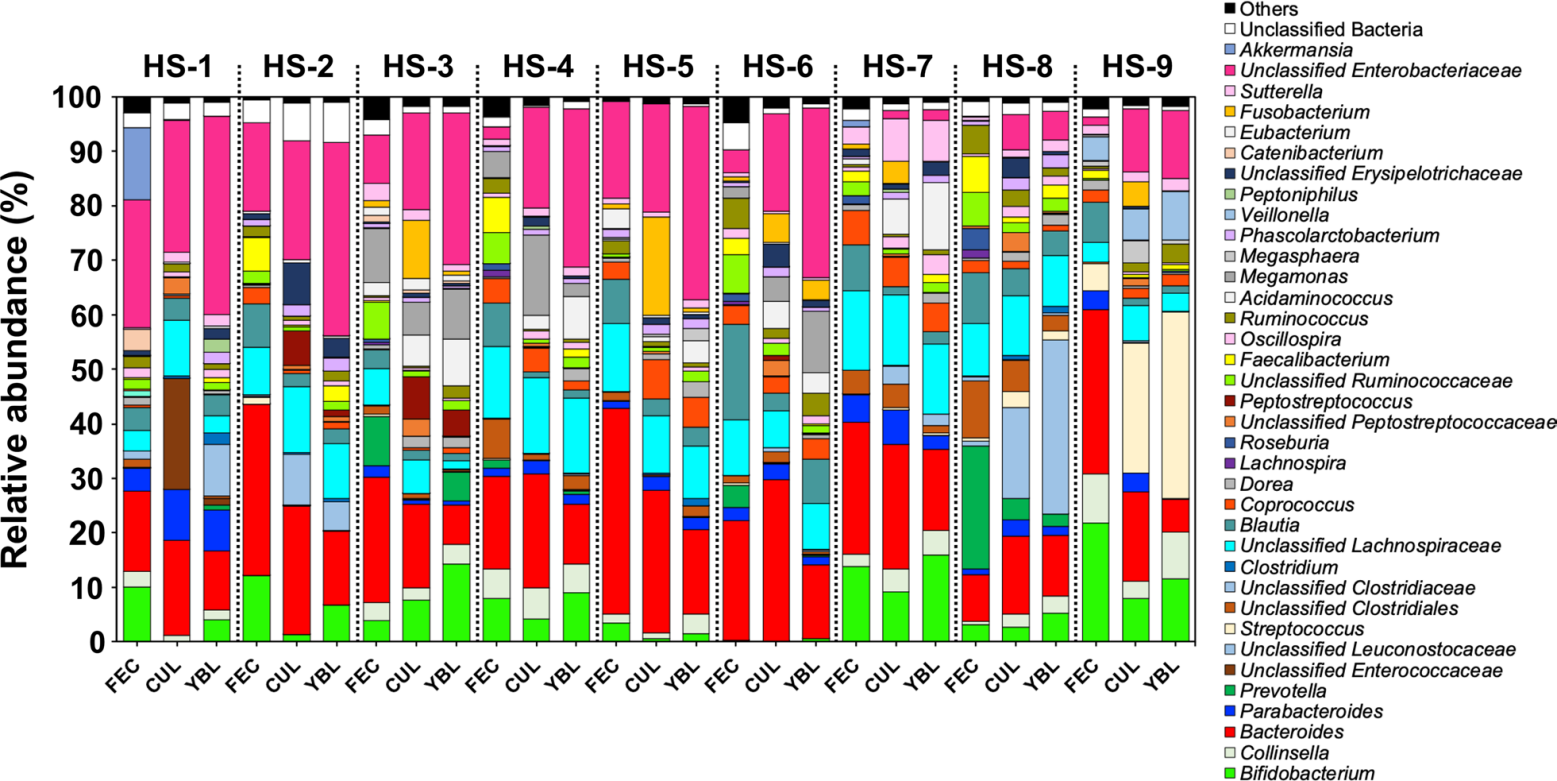
Genus-level compositional views of bacteria. The compositional views of the original fecal samples (designated as FEC), KUHIMM cultures without 1.5% YBL (CUL), and KUHIMM cultures with 1.5% YBL (YBL) after 48 h of fermentation are shown. Fecal samples were obtained from nine healthy subjects (HS-1–HS-9) and each sample was used as the inoculum to construct the corresponding KUHIMM. Genera with low abundance (< 1.0%) and low similarity (< 97%) were included in the “Others” and “Unclassified bacteria” categories, respectively

**Fig. 4:**
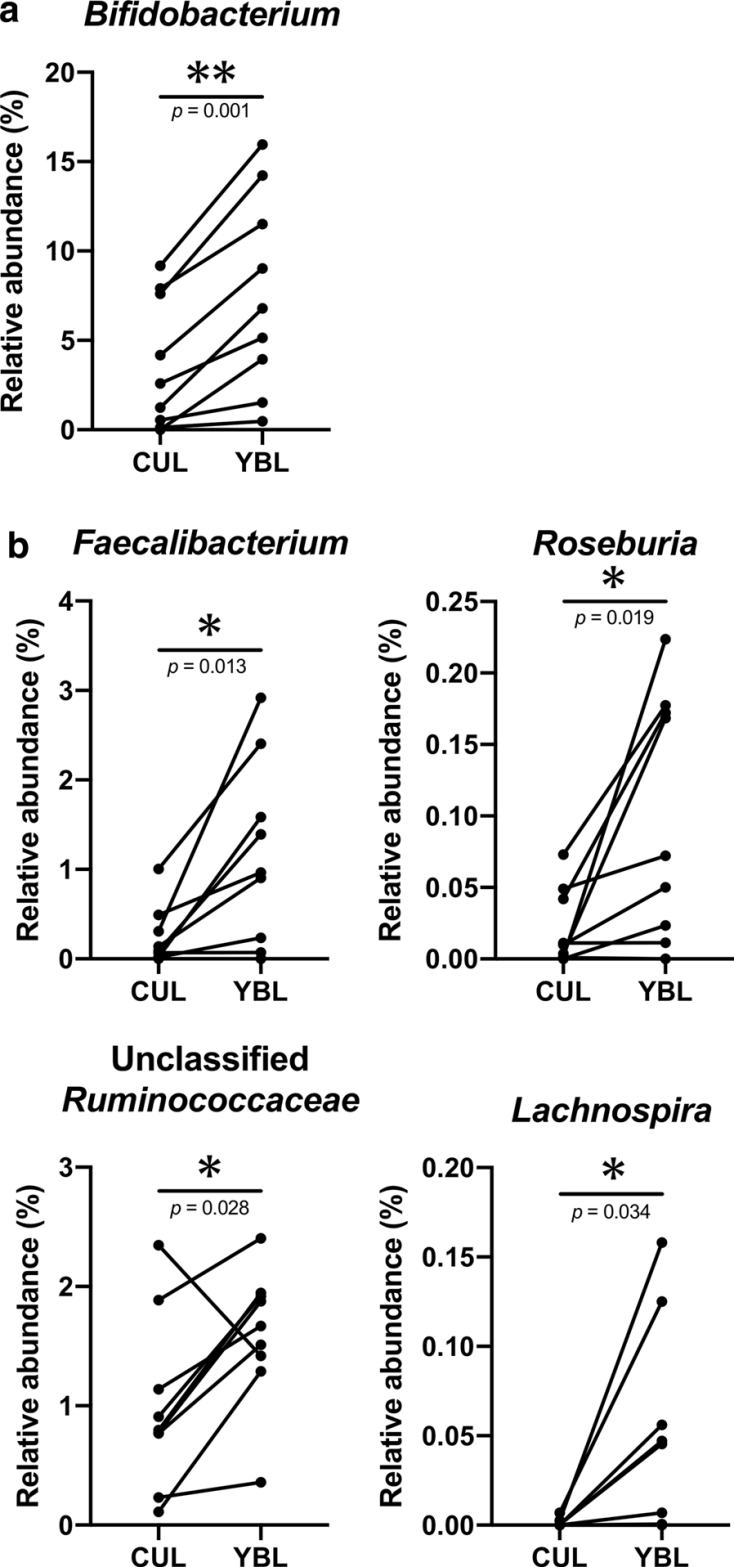
Increases in the bacterial relative abundances. The relative abundances of **a** the genus *Bifidobacterium* and **b** the genera *Faecalibacterium*, *Roseburia*, Unclassified *Ruminococcaceae*, and *Lachnospira* in the KUHIMMs with 1.5% YBL (designated as YBL) or without YBL (designated as CUL) are shown. *p < 0.05, **p < 0.01, n = 9, paired t-test

PCoA of the fecal 16S rRNA gene sequences revealed that most KUHIMMs with 1.5% YBL had microbiota compositions closer to the original fecal samples, when compared to the control KUHIMMs without YBL (Fig. 2 and Additional file 1: Figure S1). These results suggest that YBL administration affected the microbiota composition in the KUHIMM. This possibility was confirmed by performing detailed genus-level compositional analysis of the microbiota for each KUHIMM (Fig. 3). Almost all bacterial genera in the original fecal sample (designated as FEC in Fig. 3) were observed in the KUHIMM without YBL (designated as CUL in Fig. 3). Statistical analyses were carried out to compare the relative abundances of bacterial genera to all bacteria detected in the CUL and YBL samples. The most remarkable increase was observed in the YBL sample in terms of the relative abundance of commensal bacteria in the genus *Bifidobacterium*, which belongs to the phylum *Actinobacteria* (Fukuda et al. 2012), when compared to the CUL sample (Fig. 4a). In addition, increases in the proportion of other commensal bacteria in the genera *Faecalibacterium*, *Roseburia*, Unclassified *Ruminococcaceae*, and *Lachnospira*, which belong to the phylum *Firmicutes* (Lopetuso et al. 2013), were observed in the YBL sample, when compared to the CUL sample (Fig. 4b). In contrast, significant decreases in the proportions of Unclassified *Peptostreptococcaceae* and the genus *Fusobacterium* (Shang and Liu 2018) were detected in the YBL sample (compared to the CUL sample). Importantly, *Peptoclostridium difficile* (*Clostridium difficile*) and *Peptostreptococcus* spp. (Bourgault et al. 1980; Rupnik et al. 2009) in the family *Peptostreptococcaceae* and *Fusobacterium* spp. were previously reported as pathogens (Additional file 1: Figure S2).

### YBL administration enhanced butyrate production

**Fig. 5:**
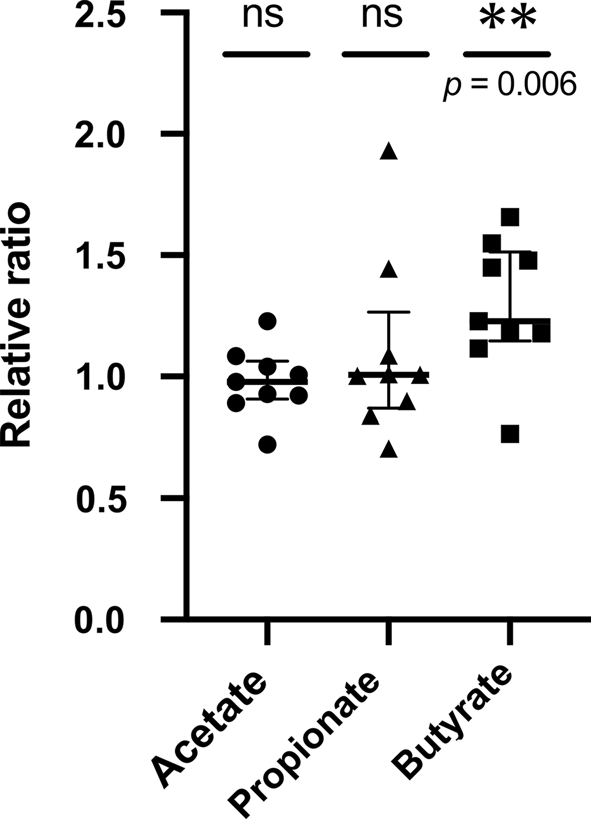
Changes in the production of SCFAs. The ratio of SCFAs concentrations in the KUHIMM cultures with 1.5% YBL to those without 1.5% YBL are shown. **p < 0.01, n = 9, using Dunnett’s test. The data shown are presented as medians and interquartile ranges (25th–75th percentiles). *ns* not significant

**Fig. 6:**
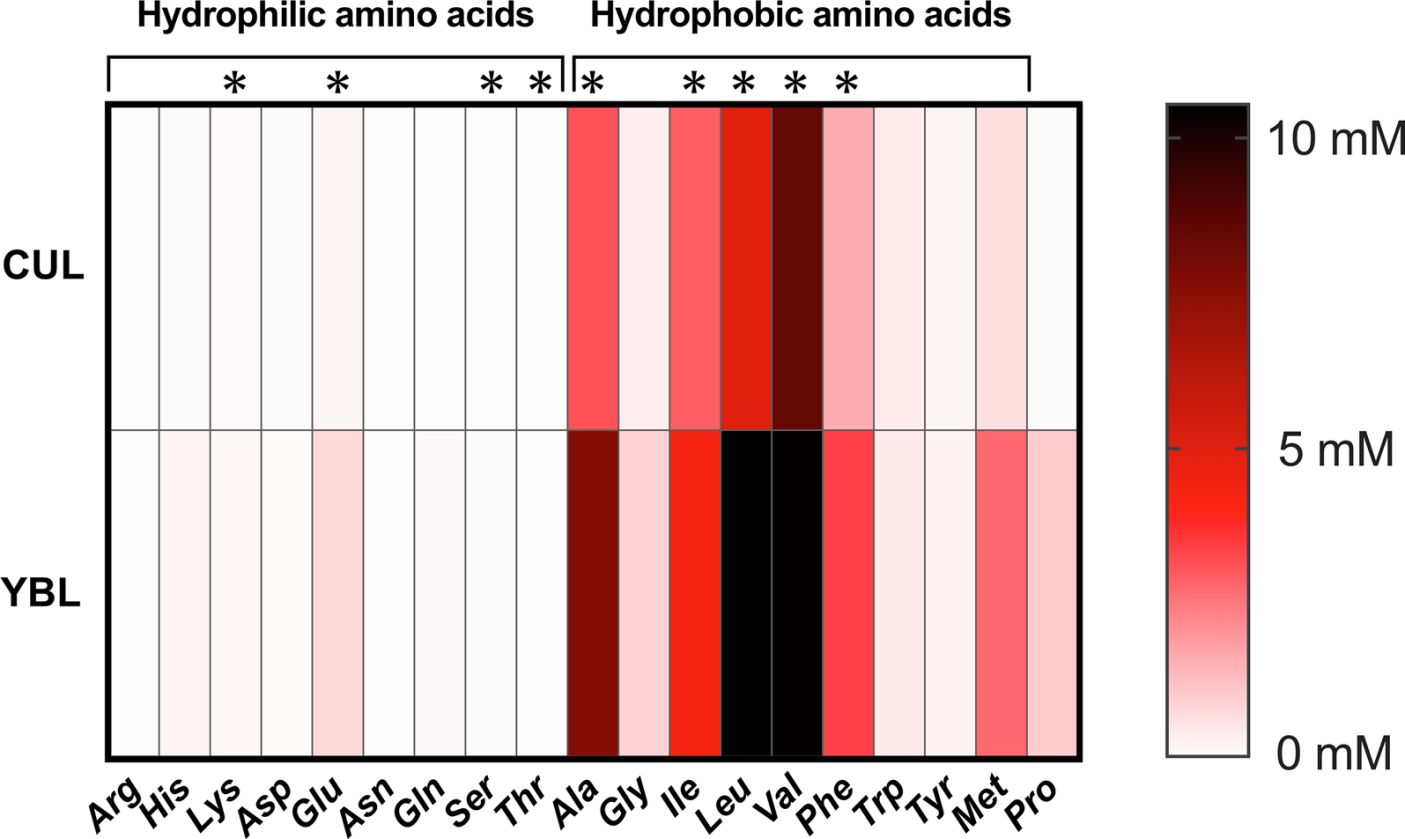
Free amino acid levels in the culture supernatants. A heatmap of the average concentrations of free amino acids in the culture supernatants of KUHIMM samples without 1.5% YBL (designated as CUL) or with 1.5% YBL (designated as YBL) is shown. Hydrophilic amino acids: *Arg* arginine, *His* histidine, *Lys* lysine, *Asp* aspartate, *Glu* glutamate, *Asn* asparagine, *Gln* glutamine, *Ser* serine, *Thr* threonine. Hydrophobic amino acids: *Ala* alanine, *Gly* glycine, *Ile* isoleucine, *Leu* leucine, *Val* valine, *Phe* phenylalanine, *Trp* tryptophan, *Tyr* tyrosine, *Met* methionine, *Pro* proline. *p < 0.05, n = 9, paired t-test

The SCFA concentrations in the KUHIMM after 48 h of fermentation were measured, and the relative ratios were calculated by dividing each concentration measured using the KUHIMM with 1.5% YBL by the corresponding concentration measured with the control KUHIMM without 1.5% YBL (Fig. 5). Although no significant changes in acetate and propionate production were detected between KUHIMMs with or without YBL, butyrate production significantly increased after YBL administration. In addition, the concentrations of 19 free amino acids were measured in the supernatants of KUHIMM cultures, with or without added YBL (Fig. 6). The concentrations of hydrophilic amino acids such as lysine, glutamate, serine, and threonine and hydrophobic amino acids such as alanine, isoleucine, leucine, valine, and phenylalanine were significantly higher in the KUHIMMs with 1.5% YBL, compared to those without 1.5% YBL.

## Discussion

We evaluated the relationships between YBL administration and the responses of human colonic microbiota. YBL exerted a bifidogenic effect by increasing the relative abundances of bacteria related to the genus *Bifidobacterium*. Previous findings showed a bifidogenic effect of barley grain (Martínez et al. 2013), although β-glucan in barley grain impacted the growth of *Bacteroides* species, but not *Bifidobacterium* species (Kristek et al. 2019). Here, we discussed which ingredients in YBL exerted a bifidogenic effect.

The YBL sample contained a substantial amount of fructans (9.4 g/100 g). Fructans consist of fructose-derived oligosaccharides and polysaccharides, and are classified as inulin (β 2 → 1 linkage), levan (β 2 → 6 linkage), and graminan (β 2 → 1 linkage and β 2 → 6 linkages) types based on their structures (Shiomi 2008; Peshev and Van den Ende 2014). Inulin-type fructans selectively stimulate the growth and/or activity of bifidobacteria because bifidobacteria possess β-fructofuranosidase, which can break down and utilize inulin-type fructans, providing a competitive advantage in a mixed culture environment (Kolida and Gibson 2007). In addition, levan-type exopolysaccharides from lactobacilli-enriched *Bifidobacterium* spp. (Bello et al. 2001) and *Bifidobacterium longum* subsp. *infantis* ATCC 15697 by hydrolyzing levan-type fructooligosaccharides (Ávila-Fernández et al. 2016). Thus, the fructans included in YBL would be expected to stimulate bifidobacterial growth.

Administering 1.5% YBL also stimulated the growth of bacteria related to the genera *Faecalibacterium*, *Roseburia*, Unclassified *Ruminococcaceae*, and *Lachnospira*. These microorganisms belong to those that produce butyrate, i.e., *Clostridium* cluster IV and *Clostridium* cluster XIVa (Duncan 2002, 2006; Ferrario et al. 2014; Vital et al. 2014). The growth stimulation corresponded with increased butyrogenesis in the KUHIMMs treated with 1.5% YBL, compared to those that were not treated with 1.5% YBL (Additional file 1: Figure S3). Recently, it was shown that butyrate producers such as *Faecalibacterium prausnitzii* and *Roseburia* spp. can consume inulin-type fructans (Falony et al. 2009; Moens et al. 2016). The consumption of inulin-type fructans by bifidobacteria provides butyrate-producing bacteria with exogenous acetate for use as a co-substrate to synthesize butyrate when growing on inulin-type fructan (Rivière et al. 2016). Another type of cross-feeding takes place between bifidobacteria that consume inulin-type fructans and produce acetate, and acetate-consuming butyrate-producing bacteria that do not degrade inulin-type fructans (Rivière et al. 2016). The occurrence of such cross-feedings between *Bifidobacterium* spp. and species of butyrate-producing bacteria was supported by our findings that increased acetate production was not detected in KUHIMM cultures with added YBL and that only butyrate production was enhanced. Therefore, our study established the bifidogenic and butyrogenic effects of YBL. Reduced bifidobacterial levels and/or butyrate producers are associated with inflammatory bowel disease and colorectal cancer (Rivière et al. 2016). YBL consumption seems to be a viable approach for counteracting such disorders.

The pH values of the culture broths with or without 1.5% YBL were 6.38 ± 0.30 or 6.70 ± 0.11, respectively, after 48 h of fermentation. The decrease in pH following YBL administration could explain the growth inhibition of bacteria related to *Peptostreptococcus* and *Fusobacterium*, considering that *Peptostreptococcus* and *Fusobacterium nucleatum* were detected at a relative higher pH (Zilm et al. 2010; Wang et al. 2012). YBL administration also increased the production of certain amino acids. Threonine is necessary for synthesis of the intestinal mucin protein backbone (Ma and Ma 2019). Branched-chain amino acids such as leucine and valine not only are essential for protein biosynthesis, but also are involved in maintaining intestinal barrier function (Ma and Ma 2019). The increased supply of such amino acids from YBL would have beneficial effects in terms of supplying nutrients and regulating the gut immune system.

## Supplementary information

**Additional file 1: Figure S1.** A distance matrix was calculated by PCoA of unweighted UniFrac distances. The distances of the PCoA plots between FEC and CUL and between FEC and YBL are referred to as “without YBL” and “with YBL”, respectively. Among the nine healthy subjects, seven PCoA plots of the KUHIMMs with 1.5% YBL were closer to those of the original fecal samples than those of the KUHIMMs without 1.5% YBL. **Figure S2.** Relative decreases in the abundances of bacteria related to Unclassified *Peptostreptococcaceae* and the genus *Fusobacterium* in KUHIMMs, cultured with 1.5% YBL (designated as YBL) or without 1.5% YBL (designated as CUL). **p < 0.01, n = 9, paired t-test. ns: not significant. **Figure S3.** Relationship between the sum of the relative abundance (%) of bacteria related to the genera *Faecalibacterium*, *Roseburia*, Unclassified *Ruminococcaceae*, and *Lachnospira* and the butyrate concentration (mM) in KUHIMM cultures with (colored circles) or without (colored triangles) 1.5% YBL, as determined after 48 h of fermentation. The different colors are related to each of the nine healthy subjects (HS-1–HS-9). The solid line and the corresponding line equation indicate the best-fit linear relationship.

## Data Availability

All raw sequence data generated in this study were deposited on the MG-RAST server (Meyer et al. 2008) (http://metagenomics.anl.gov) in a file named “Single Batch Fermentation System Simulating Human Colonic Microbiota_Young Barley Leaf Extract” under accession numbers mgm4854581.3–mgm4854607.3.

## Abbreviations

Ala: alanine
Arg: arginine
Asn: asparagine
Asp: aspartate
CUL: control culture without added YBL
FEC: fecal sample
GC–Q–MS: gas chromatography–quadrupole–mass spectrometry
Gln: glutamine
Glu: glutamate
Gly: glycine
His: histidine
HPLC: high-performance liquid chromatography
Ile: isoleucine
KUHIMM: Kobe University Human Intestinal Microbiota Model
Leu: leucine
Lys: lysine
Met: methionine
NGS: next-generation sequencing
OTU: operational taxonomic unit
PCR: polymerase chain reaction
Phe: phenylalanine
Pro: proline
SCFA: short-chain fatty acid
Ser: serine
SOD: superoxide dismutase
Thr: threonine
Val: valine
Trp: tryptophan
Tyr: tyrosine
YBL: young barley leaf extract
