# Supplementary material for: Bifidogenic and butyrogenic effects of young barely leaf extract in an in vitro human colonic microbiota model
Source: AMB Express. 2019 Nov 13;9:182. doi: 10.1186/s13568-019-0911-5 (PMC6854142; doi:10.1186/s13568-019-0911-5)
Supplement: Supplementary file 1 — Additional file 1: Figure S1. A distance matrix was calculated by PCoA of unweighted UniFrac distances. The distances of the PCoA plots between FEC and CUL and between FEC and YBL are referred to as “without YBL” and “with YBL”, respectively. Among the nine healthy subjects, seven PCoA plots of the KUHIMMs with 1.5% YBL were closer to those of the original fecal samples than those of the KUHIMMs without 1.5% YBL. Figure S2. Relative decreases in the abundances of bacteria related to Unclassified Peptostreptococcaceae and the genus Fusobacterium in KUHIMMs, cultured with 1.5% YBL (designated as YBL) or without 1.5% YBL (designated as CUL). **p < 0.01, n = 9, paired t-test. ns: not significant. Figure S3. Relationship between the sum of the relative abundance (%) of bacteria related to the genera Faecalibacterium, Roseburia, Unclassified Ruminococcaceae, and Lachnospira and the butyrate concentration (mM) in KUHIMM cultures with (colored circles) or without (colored triangles) 1.5% YBL, as determined after 48 h of fermentation. The different colors are related to each of the nine healthy subjects (HS-1–HS-9). The solid line and the corresponding line equation indicate the best-fit linear relationship. [file 13568_2019_911_MOESM1_ESM.pdf]

Additional File 1:

Submitted to *AMB Express*

Title: Bifidogenic and butyrogenic effects of young barely leaf extract in an *in vitro* human colonic microbiota model

Authors: Daisuke Sasaki[1], Kengo Sasaki[1*], Yasushi Kadowaki[2], Yasuyuki Aotsuka[2], Akihiko Kondo[1,3]

Author details:

[1]Graduate School of Science, Technology and Innovation, Kobe University, 1-1 Rokkodai-cho, Nada-ku, Kobe, Hyogo 657-8501, Japan

[2]JPD Co., Ltd., 7-98 Kita-Itami, Itami-shi, Hyogo 664-0831, Japan

[3]RIKEN Center for Sustainable Resource Science, 1-7-22 Suehiro-cho, Tsurumi-ku, Yokohama, Kanagawa 230-0045, Japan

Corresponding author:

Kengo Sasaki

Graduate School of Science, Technology and Innovation, Kobe University

1-1 Rokkodai-cho, Nada-ku, Kobe, Hyogo 657-8501, Japan

Tel: +81-78-803-6462, Fax: +81-78-803-6462, Email: sikengo@people.kobe-u.ac.jp

Figure S1.

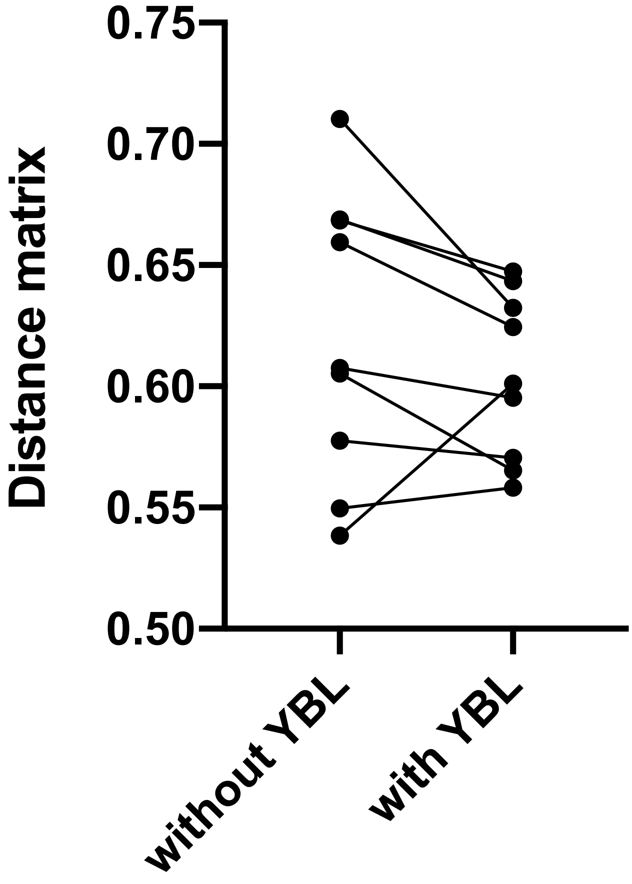

A dsistance matrix was calculated by PCoA of unweighted Unifrac distances. The distances of the PCoA plots between FEC and CUL and between FEC and YBL are referred to as “without YBL” and “with YBL”, respectively. Among the nine healthy subjects, seven PCoA plots of the KUHIMMs with 1.5% YBL were closer to those of the original fecal samples than those of the KUHIMMs without 1.5% YBL.

Figure S2.

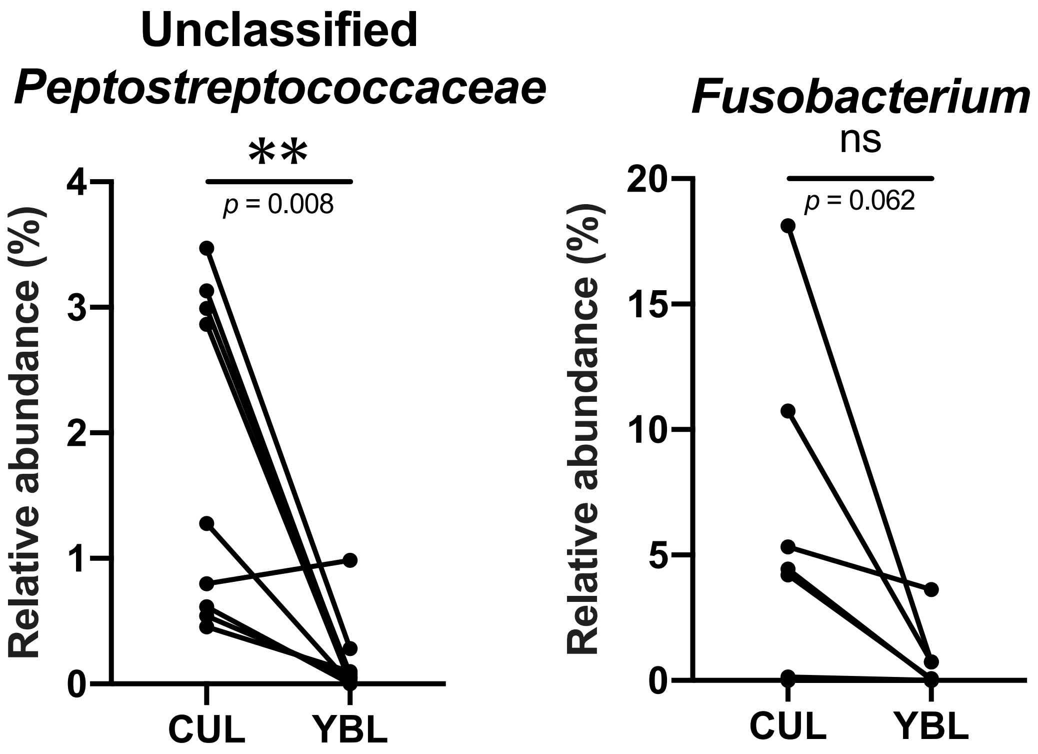

Relative decreases in the abundances of bacteria related to Unclassified *Peptostreptococcaceae* and the genus *Fusobacterium* in KUHIMMs, cultured with 1.5% YBL (designated as YBL) or without 1.5% YBL (designated as CUL). **$p < 0.01$, n = 9, paired t-test. ns: not significant

Figure S3.

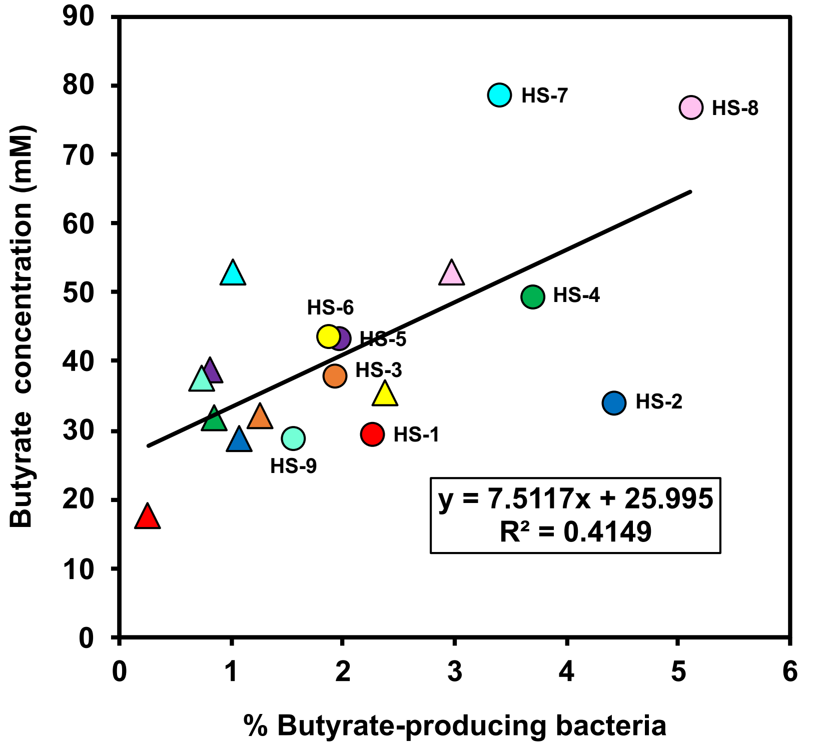

Relationship between the sum of the relative abundance (%) of bacteria related to the genera *Faecalibacterium*, *Roseburia*, Unclassified *Ruminococcaceae*, and *Lachnospira* and the butyrate concentration (mM) in KUHIMM cultures with (colored circles) or without (colored triangles) 1.5% YBL, as determined after 48 h of fermentation. The different colors are related to each of the nine healthy subjects (HS-1–HS-9). The solid line and the corresponding line equation indicate the best-fit linear relationship.
